# The AIMS approach: regulating receptivity in patient-provider vaccine conversations

**DOI:** 10.3389/fpubh.2023.1120326

**Published:** 2023-06-02

**Authors:** John Parrish-Sprowl, Angus Thomson, Rodger D. Johnson, Susan Parrish-Sprowl

**Affiliations:** Department of Communication Studies, Global Health Communication Center, Indiana University Purdue University Indianapolis, Indianapolis, IN, United States

**Keywords:** communication, process, trust, vaccine hesitancy, interpersonal communication, complexity

## Abstract

The World Health Organization named vaccine hesitancy a leading global health threat of modern time. Addressing this public health issue requires a multi-front strategy, one such strategic effort is training health care professionals to respond to reluctant patients/caregivers or those who refuse vaccines. AIMS (Announce, Inquire, Mirror, and Secure) is designed to help HCPs engaged in more productive conversations with patients/caregivers to secure trust, a key behavior leading to higher vaccination rates.

## 1. Introduction

The World Health Organization named vaccine hesitancy one of the top 10 threats to global health in 2019, and it has been redefined from “a delay in acceptance or refusal of vaccination despite availability of vaccination services” to “a motivational state of being conflicted about, or opposed to, getting vaccinated” (1, 2). While addressing the complicated problem of under-vaccination will require efforts on multiple fronts, enhancing the capacity of health care professionals (HCPs) to effectively respond to patients who refuse or are reluctant is a critical component of this mission. Research suggests HCPs are often the most trusted source of information on vaccines (3), but an escalation in antivaccination messages across various social media platforms in recent years has increased uncertainty across the general population. HCPs are confronted with the systemic effects of this trend on a daily basis as they see concerned patients/caregivers with a range of distressing emotions who have inaccurate or incomplete information, feel pressured by friends and relatives, and are struggling with how to make good decisions about their children’s health. This essay introduces a recently developed intervention framework, referred to as AIMS (Announce, Inquire, Mirror, Secure), that is designed to help HCPs facilitate a productive conversation with patients/caregivers about this important but sometimes difficult topic. AIMS is informed by scientific research from multiple disciplines, can be adapted to context and culture, and is specifically tailored to work in today’s challenging healthcare environment. Feedback from multiple trainings with HCPs from more than 20 countries suggests this approach can be a vital tool in our efforts to increase vaccine acceptance. Additionally, 93% of participants felt empowered to proactively talk about vaccination with their patients, and 80% believed the acquired skills will help their daily practice.

## 2. Background

As researchers, public health officials, and practitioners have grappled with the growing problem of addressing vaccine hesitancy or refusal over the past several years, a developing consensus has begun to emerge about what does not work. There is a recognition that the evidence base has historically been confined to research looking at the content of messages created to counter arguments leveled against vaccination (4). Gagneur et al. (5) note however, that “(t) he traditional approach of oversaturating caregivers with facts about vaccination, facts that they might not even listen to, seems to be obsolete, particularly for the new generation of caregivers who can access a lot of information on the internet” (p. 6554). Others have argued that such “data dumping” can backfire and reinforce or even strengthen hesitancy (6, 7). Increasingly, those interested in improving vaccination adherence have started to turn their attention to the process of communication and the quality of HCPs’ interactions with patients/caregivers for new insights (4). For example, Gagneur et al. (5) report an increase in caregivers’ intention to vaccinate after an educational intervention, tailored to their assessed readiness to vaccinate, delivered using motivational interviewing techniques that encouraged discussion and questions instead of offering “prescriptive and direct information.” Also utilizing principles of motivational interviewing, Leask et al. (6) offer a framework for vaccination discussions, based on caregivers’ stage of behavior change, that encourages “respectful interactions that aim to guide caregivers toward quality decisions” (p. 1). Maurici and colleagues (8) assessed “the impact of a three-day residential course on empathy and counseling abilities on the caregiver-rated level of empathy of healthcare staff working in vaccination centers in the South of Italy” (p. 1). While the question of vaccination uptake was not directly addressed in their study, they report positive results on patients perceived level of empathy for doctors and nurses.

Pfattheicher et al. (9) examined how empathy effected decision making to vaccinate in the COVID-19 pandemic. They noted that in “high-stakes” contexts, such as a global pandemic, individuals’ reactance can impede vaccination rates if people feel like their emotions are being “manipulated,” if that influence is perceived to alter their freedom and control. Their study showed that “empathy can nonetheless increase overall intention to get vaccinated” because it does not directly engage an individual’s freedom or autonomy over their decision to vaccinate or not to vaccinate (p. 6). Rather empathy can convey compassion in the exercise of reflective listening as a conversation unfolds between the patient/caregiver and provider. During an interaction the provider may inquire about discrepancies between what is perceived as the goal of a patient/caregiver and their observed behavior may help the providers adjust the conversation to directly engage discrepancy. This is the current approach of motivational interviewing (MI), which is one of the more frequently used engagement strategies for vaccine hesitant people (10). More recently, researchers Dainton and Wong (11) have argued it is “our responsibility to vaccine hesitant individuals with profound compassion,” humility, and stresses the importance of practicing empathy moving forward (p. 212).

Henrikson et al. (12) conducted a randomized trial of the impact of a physician-targeted communication training on maternal vaccine hesitancy and physician self-efficacy with 347 mothers across 56 clinics. The intervention strategy, “Ask, Acknowledge, Advise,” was “adapted from effective communication models, informed by constructs from the theory of planned behavior and based on best practices in physician-patient communication adapted to vaccine conversations” (p. 71). The 45-min training included a didactic presentation on the topics of vaccine hesitancy, provider influence on vaccine decision making, and the need to build trust with caregivers about the topic of vaccination. Trainers also facilitated discussion of videos modeling the strategy, and ways they could better manage clinic flow to improve uptake. The study found that the intervention did not reduce maternal vaccine hesitancy, nor did it improve physician self-efficacy. The authors acknowledge several challenges related to addressing vaccine hesitancy as well as limitations to their study that might account for the null findings, including uncertainty regarding the strength of the intervention. The authors note, for example, that aspects of the study implementation meant that some mothers could have seen a physician who had only partial exposure to the intervention training or was not trained at all. In a commentary on the study, Leask and Kinnersley (13) also question the brevity of the training even as they acknowledge its pragmatism given constraints on physicians’ time. They correctly observe that “[c] ommunication interventions are only effective if physicians effectively take them up … too small a dose of training will have no impact even if the intervention could work under ideal conditions” (p. 181).

Overall, a shift from a “tell and sell” message-focused communication strategy to a more relationally aware approach represents a significant development in our efforts to better address the problem of vaccine hesitancy. The seeming simplicity of using a message-focused intervention to persuade hesitant patients/caregivers to vaccinate is compelling (e.g., “just give them the facts”), but it is clearly inadequate. The studies discussed above show some promising results, but they also point to some of the difficulties we face. We must grapple with the complexity of how to better manage HCP-patient/caregiver vaccination conversations within the constraints often seen in medical settings to more effectively respond to this global health threat. There are a multitude of challenges to designing communication interventions that are both effective and pragmatically scalable. Consequently, we must not let “the perfect be the enemy of the good.” Evidence-informed approaches to vaccine conversation management that can be easily learned, practiced, remembered, and used by HCPs are needed. At the same time, we must use all available means to maximize the potential for the intervention to work. The AIMS framework is a relationship-oriented approach that has promise in accomplishing these goals. It moves us forward by offering a user-friendly vaccination conversation algorithm based on a conceptual integration of multidisciplinary scientific theory and research linking social, mental, and biological processes.

Central to understanding the potential impact of AIMS compared to other approaches is the recognition that communication is a bioactive and systemic process that has much more dimensionality than the message content of an interaction (14, 15). As we will discuss below, research shows that the quality of our interactions with one another literally shapes and is shaped by our biology, between individuals, across communities and around the globe. Expanding our understanding of how to more intentionally manage the communication ecologies within which we live and work represents a largely untapped resource in health care. From how our nervous system functions to whether or not particular genes get activated, understanding the implications of the constant interplay between talk and biology is critical for the design of effective health care interventions (16–21).

Within the health care literature generally, there is more attention being given to exploring the relationship between communication and health outcomes. For example, a number of studies have linked empathic communication by HCPs to positive health outcomes, including reducing preoperative anxiety and increasing surgical recovery and wound healing (22), fewer hospital admissions for metabolic crisis with diabetes patients (23), and faster recovery and less severe symptoms for patients with the common cold (24). In a Meta-analysis of studies on physician communication and patient adherence to treatment, (25) highlight the importance of HCP communication skills, reporting a 19% higher risk of non-adherence for patients whose physicians communicate poorly versus those who communicate well. Findings from other studies suggest interesting heuristic possibilities for health interventions. Tuck et al. (26) report the results of a study showing that being more skilled at expressing positive emotion (whether or not you actually feel it) is associated with lower cardiovascular disease risk scores. Ayling et al. (27, 28) found that having a positive mood on the day of influenza vaccination was associated with enhanced effectiveness of the vaccine in older adults. These and similar studies point to the inextricable link between social, mental, and biological processes. They highlight the potential positive impact HCPs can have on health outcomes for their patients if they are intentional in their communication, as well as the potential negative impact if they are not.

So, what does it mean to be “intentional” about communication in the design of a health care intervention and how can we maximize its benefit? AIMS moves beyond the notion of simply “being empathic” in the interaction, to thinking about how particular conversational patterns, enacted verbally and nonverbally, between the HCP and the patient/caregiver can activate neurobiological processes in both parties that help shift the communication ecology to one of receptivity rather than reactivity. It is a systemic way of thinking about the complexity of the vaccination decision process for the patient/caregiver. Within that frame, it intentionally focuses on trust-building to create a relationship that increases the possibility of a positive vaccination decision preferably during the visit, but if not, sometime in the future. In a series of in-depth interviews with new mothers, some of whom intended to vaccinate and some who did not, Benin et al. (29) found that “[t] he theme of trust in the medical profession was the Central concept that underpinned all of the themes about decision-making” (p. 1532) for both groups. The AIMS approach encourages a respectful dialog that responds to that core issue.

The science underlying AIMS has been rapidly developing over the past several decades as our technological capacity to explore the complexities of human experience has expanded. Increasingly, scholars are integrating social science and humanities understandings of human behavior with discoveries in disciplines such as neuroscience, genetics, epigenetics, neuroimmunology and others to more broadly explore the systemic nature of our world. Theory and research growing out of this trend offer novel ways to think about the interplay between mental, social/environmental, and biological processes. Cognitive neuroscientists have suggested, for example, that “[c] ognition materializes in interpersonal space” (30, p. 114). The discovery of mirror neurons, our capacity for neuroplasticity across the lifespan, and the ability for social interaction to impact us at the epigenetic level are just a few examples of recent scientific findings that are contributing to a paradigm shift in our thinking about health interventions.

The mental health disciplines have widely embraced these findings because of the implications for exploring new ways of improving mental health and overall well-being. Dan Siegel’s seminal work, the Developing Mind (31), provides a synthesis of research from multiple disciplines that informs the development of the field of Interpersonal Neurobiology which articulates a complexity-based systemic link between the mind, the embodied brain, and relationships, equating health with integration across multiple domains. Stephen Porges’ (32) work on Polyvagal Theory articulates his view of how the autonomic nervous system functions in relation to perceived external threats as well as helping us make positive social connections. It has contributed to our thinking about the importance of the body’s stress response and its systemic role in human health, including the detrimental physiological effects of sustained activation of the sympathetic nervous system. It also offers insight into how we might intentionally manage our own nervous system’s response to a situation, for example a contentious vaccination conversation with a caregiver, to both improve our capacity to respond well but also to conversationally and biologically invite the caregiver into a more receptive state. Others in the field, such as (16), Arden (33–35), have made substantial contributions that encourage us to broaden our understanding about the interplay of a range of factors that can impact overall health across the lifespan and even beyond in order to transform how we approach healthcare.

As this body of work continues to grow, we are beginning to see it applied in contexts beyond the therapy suite, such as in organizations and educational settings. Elaborating this way of thinking in healthcare generally is a natural extension and holds great potential for innovative interventions. The depth at which an HCP wishes to access this literature will vary according to individual needs and preferences. Certainly, all HCPs who are interested in ensuring their interactions with patients are supportive of positive health outcomes will likely be interested in learning approaches that science suggests would do so. To that end, it is important to develop interventions incorporating this knowledge to facilitate the kinds of conversations about vaccination and other health related behaviors that can move patients toward better decision-making. Certainly, a challenge will be how to translate the complex ideas from this research into usable and effective interventions that respond to the realities and demands of our current healthcare environment. AIMS represents an attempt to achieve that goal.

## 3. The AIMS approach to vaccination conversations

AIMS is an algorithm for a conversation between an HCP and a patient/caregiver that is designed to evoke greater psychological and emotional receptivity by intentionally activating the calming parasympathetic nervous system in both parties as a means of facilitating greater openness and trust in the relationship. Rather than a message-oriented approach that emphasizes “telling and selling,” AIMS focuses on using a conversational structure that facilitates the creation of a relational context which is more conducive to a positive vaccination decision. The process is directed by the HCP and can fit within the time constraints of the typical clinical visit.

An acronym for the structure of the vaccination conversation, AIMS stands for Announce, Inquire, Mirror, and Secure. After greeting the patient/caregiver, the HCP should take a slow, deep breath to intentionally put themselves into their most receptive mode. They should then *Announce* that it is time for the vaccination, in a friendly, non-paternalistic, matter-of-fact professional manner. Research supports such a presumptive approach because the majority will go ahead and vaccinate with that simple intervention (36, 37). This contributes to the time efficiency of the AIMS approach. Should someone agree, once they are vaccinated the HCP can complement them on making a good choice, recommend they encourage others to do so as well and proceed with the rest of the exam. However, if the patient/caregiver, pauses or expresses *any* concern or hesitation, the conversation should immediately turn to *Inquire*.

At this point, it is important that the HCP talk “with” the patient/caregiver rather than “at” them. Throughout the conversation, the HCP is encouraged to convey an attitude of curiosity and respect to even the most resistant patient/caregiver to create a relational frame that can best support a positive vaccination decision. The HCP should ask questions that elicit their reasons for hesitancy, but in ways that do not encourage them to take a position. Open-ended “how” and “what” questions tend to be more effective than “why” questions. Inquiry, rather than offering facts or reasons to vaccinate, serves many purposes and contributes to a conversation that strengthens trust in the relationship. First, it indicates that the HCP is interested in understanding what the patient/caregiver is thinking and feeling. Second, it provides the HCP with specific information that enables a tailored response to the feelings and concerns being expressed. This is more time-efficient and helps the HCP avoid bringing up concerns that the patient/caregiver had not considered on their own which can increase anxiety and reinforce hesitancy. Finally, it is an empowering opportunity for the patient/caregiver to be able to express their perspective and have it treated seriously. By engaging them, the HCP is signaling respect for the person which fosters receptivity.

Once the inquiry is responded to by the patient/caregiver, it is still not the time for the HCP to respond directly to the concerns raised. Instead, the HCP should *Mirror* the response to demonstrate both to the patient/caregiver and to themselves that they fully understand the person as the person intended it. There is a difference between understanding someone from your own perspective and doing so from theirs. They need to believe that the HCP both understands their perspective and respects them, even if they disagree with what has been expressed. In short, the person needs to “feel felt” by the HCP. This builds receptivity in the patient/caregiver and contributes to greater trust in the HCP.

The Inquiry-Mirroring process may well go through multiple iterations until the HCP has established with the patient/caregiver that understanding has been accomplished. At this point, the HCP moves to *Secure* trust. This is the point where the HCP responds to concerns with information that fits the needs of the patient/caregiver and is presented in a way that is reflective of their perspective. It should be noted that because of the focus on receptivity and the activation of the parasympathetic nervous system thus far, the patient/caregiver will be much more likely to actually take in and process information at this point in the conversation (38). If the person is still hesitant or refusing, then the HCP can say that, while in their professional opinion they disagree with the patient/caregiver, they both share a concern for the health of the patient/child. The HCP is moving to secure trust and mutual respect. This enables a future conversation where the issues can be revisited in a potentially more productive manner. Secure, then, is about the relationship, not about persuading the person regarding vaccination. Vaccination decisions, especially for the hesitant or the refuser, are complex and can involve an array of factors other than information about vaccines. This can include familial or friendship relationships, fears based on earlier trauma (such as a miscarriage), or difficulties with making decisions in general that emanate from other circumstances. Securing a relational context of caring and trust affords the best possible conditions for eventually creating a positive decision to vaccinate. In that sense, it directly responds to Leask and Kinnersley (13) call for the development of new approaches for vaccine consultation that emphasize both patient/caregiver satisfaction and positive decision to vaccinate.

Recent evidence from two study supports the usefulness of AIMS as an effective intervention to address vaccine hesitancy. Although the results of one study are limited, (39) tested AIMS to determine whether it could elicit specific vaccine favorable behaviors in caregivers. The results of the study found that in a controlled environment behaviors associated with the AIMS communication protocol were readily identified among the AIMS-trained HCPs. A more recent study of AIMS worked with 1,200 participants from over 100 countries. The researchers found that three-month post training 61% of HCPs reported increased empathy toward patients/caregivers, confidence while counseling, and increased vaccine acceptance (40). Module 3 of the AIMS training (interpersonal communication) received the highest score consistently across the five areas covering content satisfaction and delivery. Importantly, 90% (322) of participants who participated in follow-up survey (358) reported a change in their approach when dealing with caregivers, patients, and others as a result of training (40). More than two-thirds of survey respondents (358) have held conversations with patients and/or caregivers related to vaccine hesitancy and advocated for key individuals or institutions to promote the value of vaccines (40).

## 4. Conclusion

The elements of AIMS are parts of a communication process that, according to science, offer a way for HCPs and patients/caregivers to build additional strength in their relationship that can not only enhance the possibility of a positive decision to vaccinate, but can also have a number of additional health benefits. The approach can be used by different types of HCPs, thus increasing coverage, and training can take as little as 3 hours. This, combined with intentional practice by the HCP, can instantiate patterns of interaction that hold positive benefits for them and patients/caregivers. AIMS is a next generation algorithm that simplifies the application of a complex mix of science to focal conversations that research suggests are key to increasing vaccination among the hesitant and opening the door to future dialog with those who currently refuse. It represents a promising step forward in our collective effort to respond to this global health threat.

## Data availability statement

The original contributions presented in the study are included in the article/supplementary material, further inquiries can be directed to the corresponding author.

## Author contributions

JP-S, AT, and SP-S contributed to the conceptual development and writing of the original manuscript. RDJ updated, edited for clarification, and formatted the manuscript. All authors fully contributed to the manuscript and approved the submitted version.

## Conflict of interest

The authors declare that the research was conducted in the absence of any commercial or financial relationships that could be construed as a potential conflict of interest.

## Publisher’s note

All claims expressed in this article are solely those of the authors and do not necessarily represent those of their affiliated organizations, or those of the publisher, the editors and the reviewers. Any product that may be evaluated in this article, or claim that may be made by its manufacturer, is not guaranteed or endorsed by the publisher.
